# Copper doped zeolite composite for antimicrobial activity and heavy metal removal from waste water

**DOI:** 10.1186/s13065-019-0563-1

**Published:** 2019-04-01

**Authors:** Feleke Terefe Fanta, Amare Aregahegn Dubale, Dawit Firemichael Bebizuh, Minaleshewa Atlabachew

**Affiliations:** 1 Department of Chemistry, College of Natural and Computational Science, Energy and Environment Research Center, Dilla University, 419, Dilla, Ethiopia; 2 Department of Chemistry, College of Science, Bahir Dar University, P.O. Box 79, Bahir Dar, Ethiopia; 3 Blue Nile Water Institute, Bahir Dar University, P.O. Box 79, Bahir Dar, Ethiopia

**Keywords:** Wastewater, Copper-doped zeolite X, Adsorption, Heavy metals, Disinfection, Akaki river

## Abstract

**Background:**

The existence of heavy metals and coliform bacteria contaminants in aquatic system of Akaki river basin, a sub city of Addis Ababa, Ethiopia has become a public concern as human population increases and land development continues. Hence, it is the right time to design treatment technologies that can handle multiple pollutants.

**Results:**

In this study, we prepared a synthetic zeolites and copper doped zeolite composite adsorbents as cost effective and simple approach to simultaneously remove heavy metals and total coliforms from wastewater of Akaki river. The synthesized copper–zeolite X composite was obtained by ion exchange method of copper ions into zeolites frameworks. Iodine test, XRD, FTIR and autosorb IQ automated gas sorption analyzer were used to characterize the adsorbents. The mean concentrations of Cd, Cr, and Pb in untreated sample were 0.795, 0.654 and 0.7025 mg/L respectively. These concentrations decreased to Cd (0.005 mg/L), Cr (0.052 mg/L) and Pb (bellow detection limit, BDL) for sample treated with bare zeolite X while a further decrease in concentration of Cd (0.005 mg/L), Cr (BDL) and Pb (BDL) was observed for the sample treated with copper–zeolite composite. Zeolite X and copper-modified zeolite X showed complete elimination of total coliforms after 90 and 50 min contact time respectively.

**Conclusion:**

The results obtained in this study showed high antimicrobial disinfection and heavy metal removal efficiencies of the synthesized adsorbents. Furthermore, these sorbents are efficient in significantly reducing physical parameters such as electrical conductivity, turbidity, BOD and COD.

## Introduction

Rapid population growth and the consequent increase in anthropogenic activities have resulted in high demand for scarce water resources, generation of large volumes of wastewater requiring treatment technologies capable of removing multiple pollutants [1]. Major pollutants of concern include pathogenic microorganisms and persistent heavy metals (like Pb, Cr and Cd), which are harmful to humans when they exceed permissible limits. As per the World Health Organization’s (WHO) guideline, the maximum permissible limits of these pollutants in drinking water are 0 CFU/mL for faecal coliforms, 0.003 mg/L for Cd, 0.01 mg/L for Pb and 0.05 mg/L for Cr [2–4].

Addis Ababa, the capital city of Ethiopia, has many rivers and tributaries. Among which, Akaki Rivers (Little Akakki and bigger Akaki rivers) are those rivers which receives polluted water from several tributaries such as Kebena, BancheYeketu, Kortame, Bulbula, LequSoramba, kotebe and Fincha rivers etc. [5]. These river tributaries are becoming an environmental concern in Addis Ababa city and its vicinity areas, where (more than 40% of large and medium scale manufacturing industries are located and some of these industries discharge their effluents to these rivers without being properly treated [5, 6]. As a result, Akaki river is believed to be highly polluted chemically, physically and bacteriologically, and as a result the river water is unfit for domestic, industrial, commercial and agricultural uses [5]. Therefore, those industries discharging their effluents to this river and river tributaries require more treatment technologies that can handle multiple pollutants [7].

Among the various treatment technologies, ion exchange and adsorption are feasible and cost effective to remove heavy metals using an exchanger/sorbent with high selectivity for the target metal [8]. In the last few decades, sorbents for the treatment of heavy metals contamination have been investigated. Numerous waste biomass sources such as rice husk, saw dust, tea and coffee waste, orange peel peanut shells, activated carbon, dry tree leaves and barks, papaya seed, egg shell and coconut leaf powder were used as adsorbent for the removal of heavy metals from various waste water sources [9–12]. However, these adsorbents have several limitations such as stability, reusability and others.

An alternative to the natural products, low-cost zeolite sorbents, which have unique ion exchange and sorption properties, have been investigated as candidates for cost-effective removal of heavy metals from waste solution. Synthetic, natural and modified zeolites have been tested [1, 8, 13]. However, the modified and synthetic zeolites were found to be advantageous in terms of purity, uniform pore size and better ion exchange abilities as compared to natural zeolites [8].

Regarding microbial disinfection, several physical and chemical methods (chlorination, UV treatment, treatment with ozone, membrane filtration etc.) have been reported. Although these techniques provide water of high microbiological quality, they have several limitations such as expensiveness, formation of byproducts, requirement of pre-treatment step, and/or being resisted by some microbials [14]. Thus, as an alternatives strategy, the application of metal nanoparticles for water disinfection and treatment had been reported by many researchers. The metallic nanoparticles are most promising for disinfection as they exhibit high anti bacterial properties due to their large surface area to volume ratio. Copper in the form of nanoparticle has good bactericidal activity [15]. Hence copper has been immobilized on different inorganic support materials for a wide range of antimicrobial applications including water and wastewater disinfection [15–18]. There is also a report showing the bactericide effect of the Mexican natural zeolites modified with silver ions in the treatment of wastewater [13]. However, there is no report showing the simultaneous heavy metal and microbial removal efficiency of unmodified and modified synthetic zeolite.

Thus, this study was aimed to demonstrate the simultaneous removal effect of copper doped and unmodified synthetic zeolite for decreasing the heavy metals, biological load and some physical parameters (BOD, COD, turbidity and electrical conductivity) in wastewater treatment processes.

## Methods/experimental

### Chemicals and reagents

All chemicals used were analytical grades. Two kilogram of Kaolin sample was collected from Hadiya zone Balesa district, Ethiopia. CuSO_4_ as precursor for loading copper, deionized water for washing the adsorbent, HNO_3_ and NaOH for pH adjustment, ascorbic acid as reductant were used in the experiment.

### Instruments and apparatus

Inductively coupled plasma atomic emission spectrometry (ICP-AES) Perkin Elmer 4300 DV model from USA was used to quantify the mass of copper dissolved in the leach liquor after adsorption experiment. Flame atomic absorption spectroscopy (FAAS) equipped with deuterium background corrector and air-acetylene flame system was used to quantify the heavy metals before and after absorption; X-ray diffraction (XRD), Fourier-transform infrared radiation (FTIR) and autosorb IQ automated gas sorption analyzer were used to characterize the adsorbent; pH meter, turbidity meter (VSI-13N NEPHELOMETER), conductivity meter (1153 JENWAY) were used to measure physico-chemical parameters of the effluents.

### Waste water sampling and study area description

The Akaki catchment is located in central Ethiopia along the western margin of the Main Ethiopian Rift. The catchment is geographically bounded between 8°46′–9°14′N and 38°34′–39°04′E. Composite samples were collected in 2 weeks interval during September–October 2017 and analyzed for biochemical oxygen demand (BOD), chemical oxygen demand (COD), temperature, turbidity, conductivity, pH and total coliforms. The concentrations of the heavy metals, Cd, Pb and Cr were determined using FAAS before and after treatment. The large particles from the effluent sample were removed through sedimentation and stored at 25 °C in a dark place in acidic medium pH 2 using 1.0 M HCl solution.

### Digestion of waste water sample

Waste water samples were digested with HNO_3_ and HClO_4_ in the ratio of 3:1. The samples were digested on a hot plate at a temperature of 93 °C for 2 h following a method reported by Idera et al. [19].

### Instrument calibration

Calibration curves were prepared to determine the concentration of the metals in the untreated and treated effluent sample solution. The FAAS instrument was calibrated using four series of working standards (0.1, 0.5, 1.0, 1.5 mg/L). For the three metals, a correlation coefficient of 0.9998 was obtained.

### Synthesis of zeolite and copper doped zeolite

Zeolite X was synthesized following the method which was originally optimized in our research group. In brief, the kaolin was washed with distilled water several times to remove the impurities and dried in an open air for about a week. The dried kaolin sample was ground with mortar and pestle and then sieved with 0.25 mm mesh size sieve. Metakaolin was synthesized by calcinations of kaolin in muffle furnace at 900 °C for 2 h. About 20 g of the synthesized metakaolin was treated with 15 mL of concentrated HCl and allowed to settle for 6 h. The metakaolin suspension was added to NaOH solution to 250 mL borosilicate flask to form a gel, and shaken on an orbital shaker at 270 rpm until equal distribution of NaOH solution was obtained. To develop the zeolite framework, the homogenized sample was allowed to crystallize at 50 °C for 24 h in water bath and then dried in the oven. The dried sample was washed with deionized water to bring the pH 9, dried, powdered and crushed to uniform pore size. The copper doped zeolite was prepared by ion-exchange method. About 10 g of the synthesized zeolite X was treated with 0.1 M of copper sulphate (CuSO_4_) followed by treatment with 0.2 M ascorbic acid. The resulting Cu–Zeolite was dried at 70 °C overnight, ground using mortar and pestle and characterized for adsorption experiment. Here after, for clarity, the synthesized zeolite and copper doped zeolites are named as Z and CuZ.

### Characterization

The prepared zeolite X and copper doped zeolite X were characterized by X-ray diffraction (XRD), Fourier-transform infrared radiation (FTIR), inductively coupled plasma atomic emission spectrometry (ICP-AES) and autosorb IQ automated gas sorption analyzer. X-ray diffraction (XRD) patterns were acquired with a D2 phaser XRD-300 W, with measurements taken using Cu Ka radiation at 40 kV and 100 mA. The X-ray patterns was recorded a linear silicon strip ‘Lynx Eye’ detector from 10° to 70° at a scan rate of 0.1° min^−1^. An FT-IR spectrometer (model: Nicolet NEXUS-670) with transmission mode was used to obtain the FTIR spectra. Each sample in KBr was scanned (64 times) from 4000 to 400 cm^−1^ with a resolution of 4 cm^−1^. The copper content in the zeolite, copper modified zeolite and leach liquor samples was measured using ICP-AES Perkin Elmer 4300 DV model from USA. The specific surface area of the zeolite X and copper doped zeolite X were obtained from N_2_ adsorption/desorption data measured at 77.4 K using autosorb IQ automated gas sorption analyzer. The samples were degassed at 50 °C for 3 h prior to N_2_ adsorption. The specific surface area of the sample was calculated by using the multiple-point Brunauer–Emmett–Teller (BET) method in the relative pressure range (P/Po) of 0.05–0.3 [20].

The loading extent of copper into zeolite X was studied using ASTM D4607-94 method. Iodine number was calculated by investigating the adsorption of iodine from solution using 0.1 N standardized iodine solution and the titrant sodium thiosulfate (0.1 N). The milligrams of iodine adsorbed by 1.0 g of zeolite X when the iodine concentration of the filtrate is 0.02 N was calculated.

### Batch adsorption experiment

Batch equilibrium experiments were carried out to find the optimum adsorbent dose, contact time and initial metal concentration. About 0.5–3.0 g of adsorbent (zeolite X and copper-doped zeolite X) was added into synthetic solution of each metal and stirred for 4 h and then kept for 24 h until equilibrium could achieve and filtered. The experiment was carried out in duplicate. The optimum mass of adsorbent was obtained by plotting the percentage removal versus the mass of adsorbent. The effect of contact time in removal of Cd, Cr and Pd using zeolite X and copper doped zeolite X is studied in a contact time range of 30 to 105 min. Removal of metal ions was also carried at different initial concentration of metals (1–25 mg/L).

### Determination of Cd, Cr and Pb in waste water before and after treatment with zeolite X and copper doped zeolite X

Following the optimized procedure, 2 g of bare zeolite X or copper doped zeolite X was added to 50 mL of the composite waste water containing the Cd (0.795 mg/L), Cr (0.658 mg/L) and Pb (0.696 mg/L) metals (at pH 7.5). The solution was shaken for 60 min, filtered using Whatman filter paper, acidified to pH < 2 and stored in refrigerated prior to AAS analysis.

$$\% {\text{ metal removal efficiency}} = \, \left[ {\left( {{\text{C}}_{\text{i}} - {\text{ C}}_{\text{f}} } \right)/{\text{C}}_{\text{i}} } \right] \times 100$$

where C_i_ and C_f_ are the initial and final metal concentrations respectively.

### Bacteriological analysis

The adsorbent’s microorganism removal efficiency was tested by taking 100 mL of the river water sample before and after treatment. The number of total coliform was counted by using a magnifying glass lens. Total coliform count of waste water samples were determined by plate count method using Methylene Blue Agar and coliform colonies were enumerated and expressed as total coliform/100 mL of water samples.

### Turbidity reduction test

Turbidity of the waste water sample was tested by using portable turbidity meter. Turbidity removal efficiency of the zeolite X and copper–zeolite X composite were evaluated and compared. Turbidity of the water samples was measured relative to the turbidity of distilled water having turbidity 2NTU. The turbidity reduction was calculated by the following formula [21].

$$Turbidity \;removal \;\left( \% \right) = \frac{turbidity \;of\;source \;waste\, water - turbidity\; of\;treated\;waste\;water}{turbidity\;of\;source\;waste\;water} \times 100\%$$

### Dissolved oxygen, biochemical oxygen demand and chemical oxygen demand test

Organic pollutants in wastewater in general consist of proteins, carbohydrates and fats and oils; at approximately 50, 40 and 10%, respectively. Priority pollutants, surfactants, and emerging contaminants represent trace organic pollutants in wastewater. Dissolved oxygen (DO), biochemical oxygen demand (BOD) and chemical oxygen demand (COD) considered as the most practical indicators of water organic pollutants quality [22]. The treated and untreated wastewater samples were analyzed for DO, COD and BOD, according to standard methods for examination of water and wastewater [23].

### Conductivity and pH test

Conductivity and pH of the source river water and the treated water through zeolite X and copper doped zeolite X were measured using conductivity meter and pH meter respectively.

## Results and discussion

### Method of detection limit (MDL) and method validation

Before quantifying the metals in the water samples, the method detection limit of the FAAS was evaluated by analyzing six blank samples. It was found that the method detection limit of Pb, Cd and Cr was 0.07 mg/L, 0.03 mg/L and 0.04 mg/L respectively. The efficiency of the digestion procedure and analytical method (FAAS) was checked by adding known concentration of each metal in waste water sample. The spiked and the non spiked samples were digested and analyzed in similar conditions. Thus, the efficiency of the optimized procedure was checked by spiking 1.0 mL of 100 mg/L of Cr, 12.5 mg/L of Cd and 50 mg/L of Pb into a 50 mL of river water sample. As shown in Table 1, the results of the percentage recovery for the studied metals in Akaki river sample lie within the acceptable range. Therefore, this verifies that the optimized digestion procedure was valid for the analysis.

**Table 1:** Recovery test for the optimized procedure for heavy metals analysis

Elements	Conc. in sample (mg/L)	Amount added (mg/L)	Conc. in spiked sample (mg/L)	Recovery (%)
Pb	0.696	2.00	2.57	93.7
Cd	0.795	0.25	1.03	94.0
Cr	0.658	1.00	1.65	99.2

### Copper loading into zeolite X

Copper doped zeolite X was prepared by ion-exchange method. Iodine adsorption number is the most fundamental parameter used to identify pore size of most adsorbents. It is used to measure the activity level (higher degree indicates higher activation), often reported in mg/g (with typical range of 500–1200 mg/g) and the micropores content of the copper doped zeolite X (values > 0 to 20 Å, or up to 2 nm) by adsorption of iodine from solution. The loading extent of copper into zeolite X and their corresponding iodine number was shown in Table 2. Zeolite X doped with 6.3 g copper provided maximum iodine value (641.7 mg/g) indicating good adsorption capacity with high porosity and high surface area. Adsorption capacity of copper doped zeolite increases with increasing the amount of copper loaded from 0.63 to 6.3 g. However, further increase in copper loading beyond 6.3 g, the adsorption iodine value decreases, indicating the presence of less active sites for adsorption. Thus, based on the determined iodine value above and in Table 2, the optimum amount of copper loading into the zeolite framework was 6.3 g. Therefore, we fully characterized both undoped zeolite X (i.e. just zeolite X) and copper doped zeolite X (zeolite X loaded with 6.3 g of copper).

**Table 2:** Iodine value and mass of copper loaded in modified zeolite

Sample	Loaded copper (g)	Iodine value (mg/g)
1	0.63	478.4
2	6.3	641.7
3	63	584.4
4	126	552.9
5	189	426.2

### Crystallinity studies of adsorbent

**Fig. 1:**
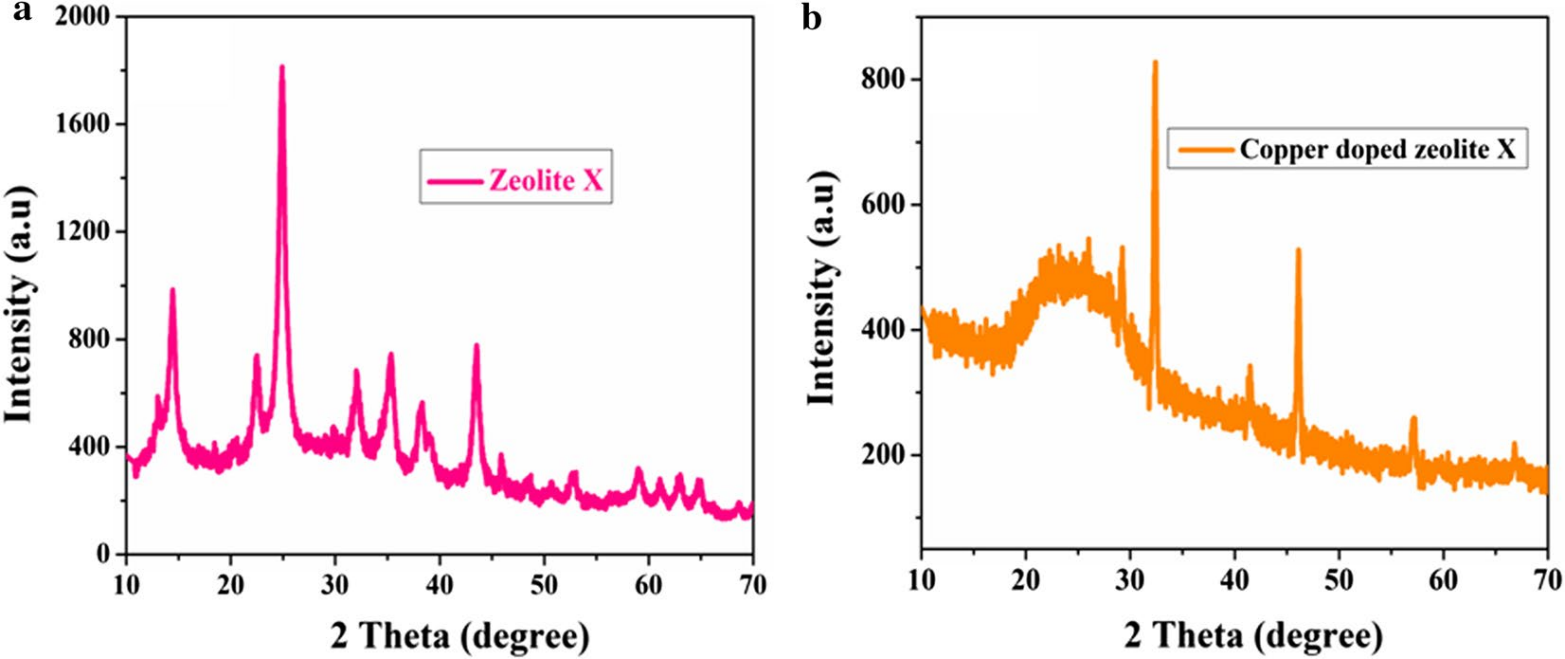
XRD patterns of **a** zeolite X and **b** copper doped zeolite X

To investigate the crystal structure and phase transformation of the synthesized zeolite X and copper doped zeolite X, we carried out XRD measurements of the samples. Figure 1 shows XRD patterns of zeolite X and copper doped zeolite X. As clearly seen in Fig. 1a, a strong diffraction peaks located at 2 Theta values of 14.3, 22.4, 24.5, 31.9, 35.3 and 43.5 are characteristics and matching well with the diffraction patterns of zeolite X peaks (JCPDS card number: 01-070-2168), indicating the profound crystalline nature of the prepared zeolite X. The XRD patters of copper doped zeolite X, shown in Fig. 1b, revealed a slight shift in the peak positions to lower degree or higher d-spacing (e.g. peak at 2*θ *= 29.4, 32.7 and 41.8) and appearance of new emerging peaks (e.g. at 2*θ *= 41.8 and 57.1) compared with XRD patterns of zeolite X (Fig. 1a). The weak intense peaks at 2*θ *= 14.3 was disappeared while the more intense peak at 2*θ *= 24.5 becomes broader after incorporation of copper into the zeolite X. A decrease in the intensities of some diffraction peaks can be also observed, but the XRD analysis of the copper doped zeolite X shows excellent crystalline nature. The overall finding from XRD pattern suggests the incorporation of copper into the zeolite X framework.

**Fig. 2:**
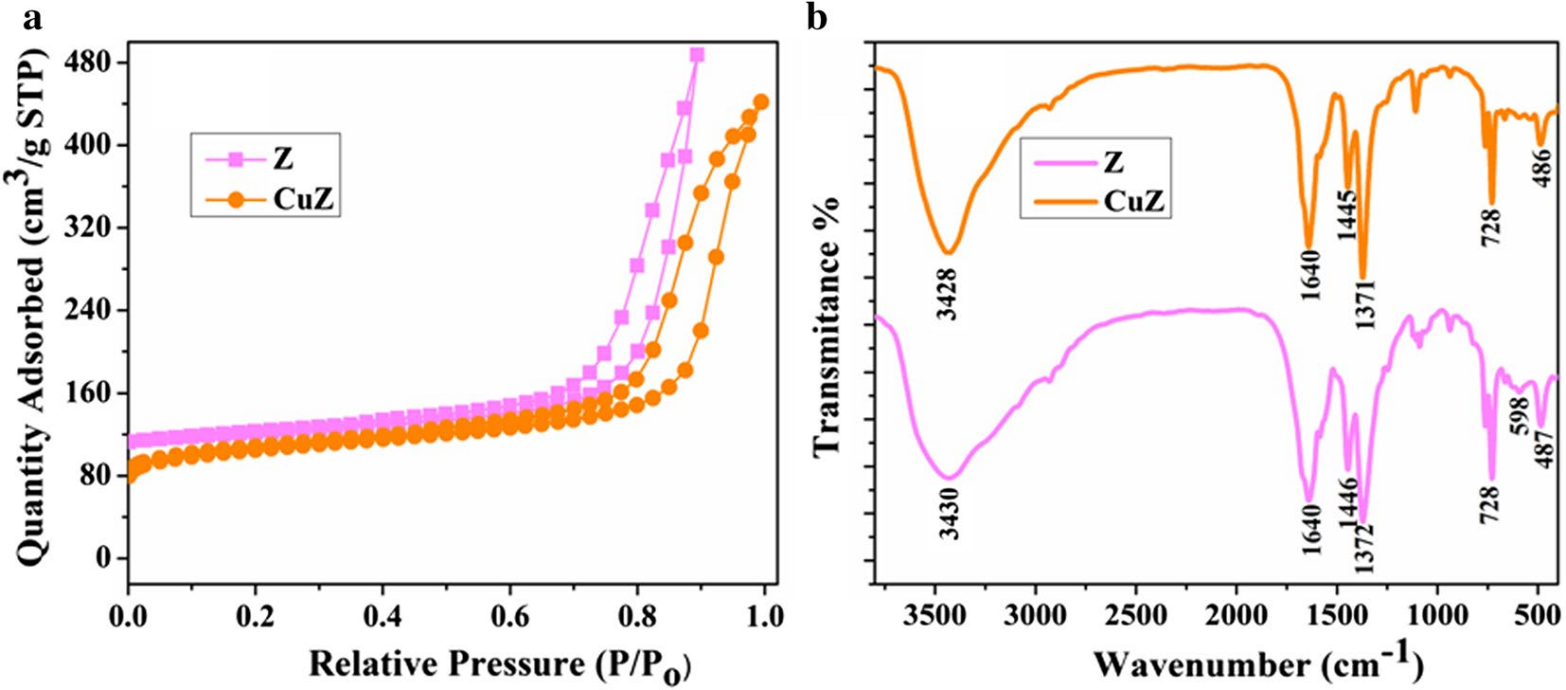
**a** N_2_ adsorption–desorption isotherms and **b** FTIR spectra of as prepared zeolite X and copper doped zeolite X

The surface area of the prepared zeolite X and copper doped zeolite X samples were investigated by measuring the nitrogen adsorption and desorption isotherms. Figure 2a shows the nitrogen adsorption–desorption isotherms of prepared zeolite X and copper doped zeolite X samples. The shape of the isotherms for both samples can be categorized to type V isotherm (i.e. based on IUPAC recommendation) with H4 hysteresis loop, indicating the existence of mesopores character of the samples. Another uptake appears at high relative pressure (P/P0 > 0.85) for both samples are attributed to the filling of macropores formed by the packing of aggregated particles [24]. The determined BET surface area of the zeolite X was found to be 460 m^2^/g, which is comparable to that of literature reported before [25]. However, the BET surface area of zeolite X was decrease to 435 m^2^/g after copper loading (i.e. S_BET_ for copper doped zeolite X sample), indicating the successful incorporation of copper into the zeolite X frameworks. The decreased in surface area is attributed to the fact that Cu are embedded in the pores of zeolite resulting partial blocking of the zeolite pore access by copper, leading to a reduction in total specific surface area. The textural properties of the samples are summarized in Table 3. Cu loading did not brought significant effect on pore volume compared with zeolite X. However, the effect of Cu loading is more pronounced in decreasing the micropore volume. That is, for zeolite X sample, the micropore volume represents 94% of pore volume, while the value drops to 81% for the copper doped zeolite X sample.

**Table 3:** Textural properties of zeolite X and copper doped zeolite X samples

Sample	S_BET_ (m^2^/g)	Pore volume (cm^3^/g)	Micropore volume (cm^3^/g)
Zeolite X	460	0.214	0.201
Copper doped zeolite X	435	0.202	0.164

To determine the structural information of the prepared zeolite X and copper doped zeolite samples, we also carried out measurement of FT-IR spectra of the prepared zeolite X and copper doped zeolite X in the region from 400 to 4000 cm^−1^ in KBr using FTIR spectrometer. Figure 2b displays the FTIR spectra of zeolite X and copper loaded zeolite X. As clearly seen in Fig. 2b, the IR spectra of zeolite X and copper doped zeolite X are quite similar to each other except that shifts of most vibrations to slightly lower frequency after Cu loading. The presence of a broad band at 3430 cm^−1^ in zeolite X sample can be ascribed to the O–H stretching, and the band shifted towards a slightly lower frequency of 3428 cm^−1^ for copper doped zeolite X sample. This might be due to a decrease in O–H bond strength. The bending vibrations of the O–H at 1640 cm^−1^ showed insignificant change in position by both samples. The peaks located at 1446 and 1372 cm^−1^ are assigned to the O–NO stretching vibrations. The T–O asymmetric stretching are observed at 938 cm^−1^ whilst the symmetric stretching of T–O (T=Si or Al) due to the internal vibrations of the zeolite X framework are observed at 728 cm^−1^. The absorption bands located at 598 and 487 cm^−1^ are assigned to the vibrations associated with the double six rings (D6R) that connect the sodalite cages and the internal vibrations due to the bending of the T–O tetrahedral, respectively. We observed a slightly shift in vibrations to lower frequency by copper doped zeolite, indicating the successful loading Cu into zeolite frameworks. This is in good agreement with our XRD and BET results.

### Optimization parameters for removal of metals (using synthetic solution)

#### Optimization of absorbent dose (zeolite X and copper doped zeolite X)

**Fig. 3:**
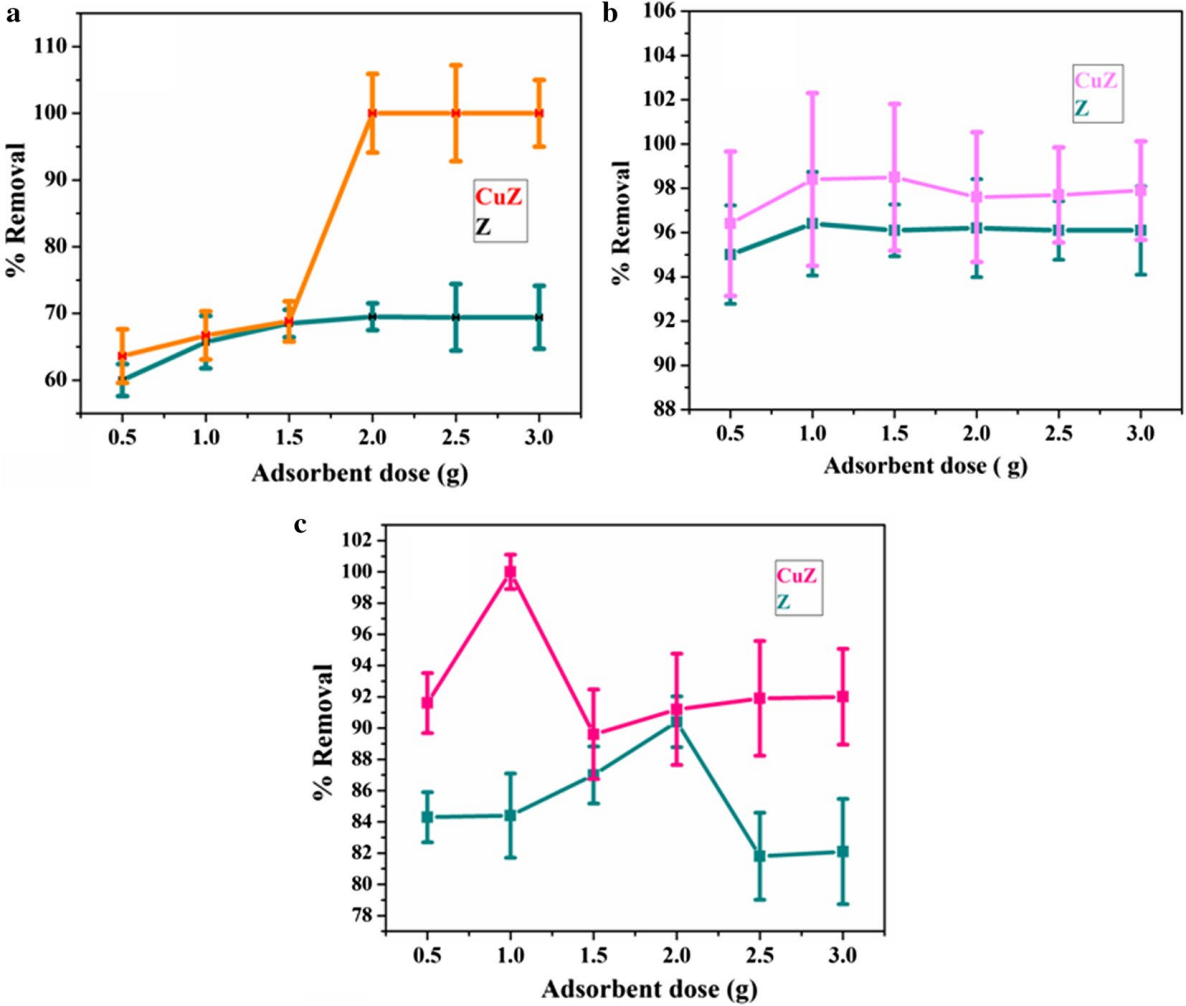
Dose effect of zeolite X and copper doped zeolite X for removal of Pb (**a**), Cd (**b**) and Cr (**c**)

The effects of zeolite X and copper doped zeolite X dosages on the removal of metal ion are illustrated in Fig. 3. The adsorbent dosage was varied from 0.5 to 3 g/25 mL for both adsorbents. The concentration of Pb, Cd and Cr before treatment were 0.696, 0.795, 0.658 mg/L respectively. At the same operating conditions (stirring speed, initial pH and temperature), the concentration of Pb, Cd and Cr were decreased to 0.214, 0.029 and 0.063 mg/L using zeolite X treatment while to BDL (bellow detection limit), 0.012 and BDL mg/L using copper doped zeolite treatment. It is observed from Fig. 3, that the removal efficiency of adsorbents generally improved with increasing the amount of zeolite X and copper doped zeolite X. This was as expected because the higher dose of adsorbent in the solution, the greater availability of exchangeable sites for the ions. At the same adsorbent dose, copper doped zeolite X has better removal efficiency for Pd, Cd and Cr metals (Fig. 3). In this finding, the optimum adsorbent dose of Pb, Cd and Cr were 2 g, 1 g, 2 g for zeolite X and 2 g, 1.5 g and 1 g for copper doped zeolite respectively (Fig. 3). At optimum adsorbent dosage the percent removal efficiency for Pd, Cd and Cr metals using zeolite X is (69.5, 96.4 and 90.4%) and for copper doped zeolite X is (100, 98.5 and 100%) respectively. As shown in Fig. 3a, at low dosage, zeolite and copper modified zeolite showed similar performance towards removal of lead. This might be due to scarcity of available enough active sites, further indicating copper modification did not bring significant change in performance at low dosage.

#### Optimization of absorbent contact time

**Fig. 4:**
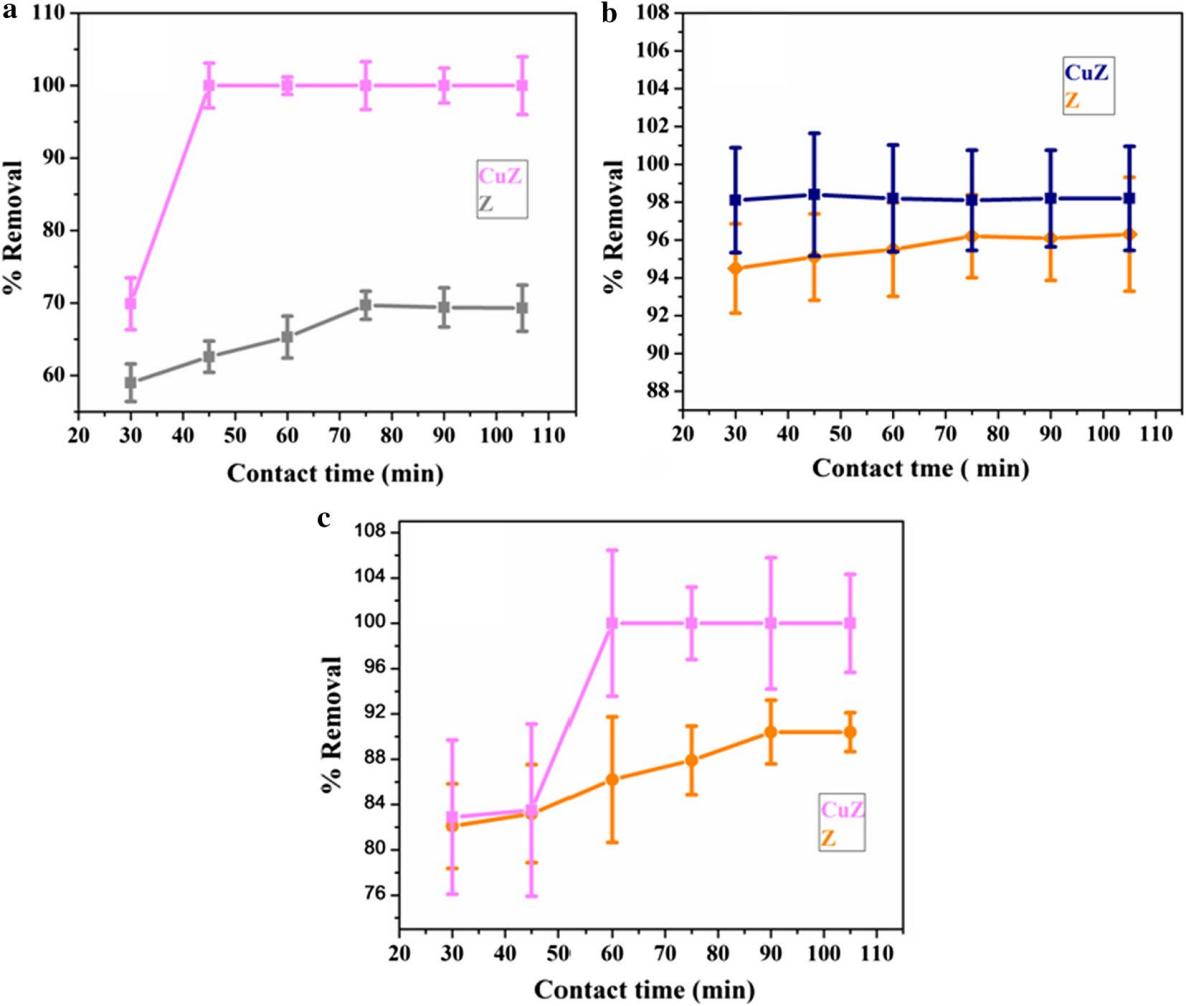
Effect of contact time on the removal of Pb (**a**), Cd (**b**) and Cr (**c**) by zeolite X and copper doped zeolite X adsorbent

The effect of contact time on the percentage removal of each metal was also studied in duplicate analysis by using optimum adsorbent dosage (zeolite X and copper doped zeolite X). The applied time intervals were 30, 45, 60, 75, 90, and 105 min. As shown in Fig. 4, increasing the contact time increases the percentage removal of each metal until the system reaches the adsorption equilibrium. From this study, it is found that the optimum contact time revealing maximum percentage removal of Pd, Cd and Cr were 75, 105, 90 min and 45, 45, 60 min for zeolite X and copper doped zeolite respectively. In all the cases, copper doped zeolite X provided maximum percentage removal of Pd, Cd and Cr (100, 98.4 and 100%) in short period of time than bare zeolite X (69.7, 96.3 and 90.4%). This indicates, copper doped zeolite X had more active sites than bare zeolite X.

### Effect of the initial metal ion concentration

**Fig. 5:**
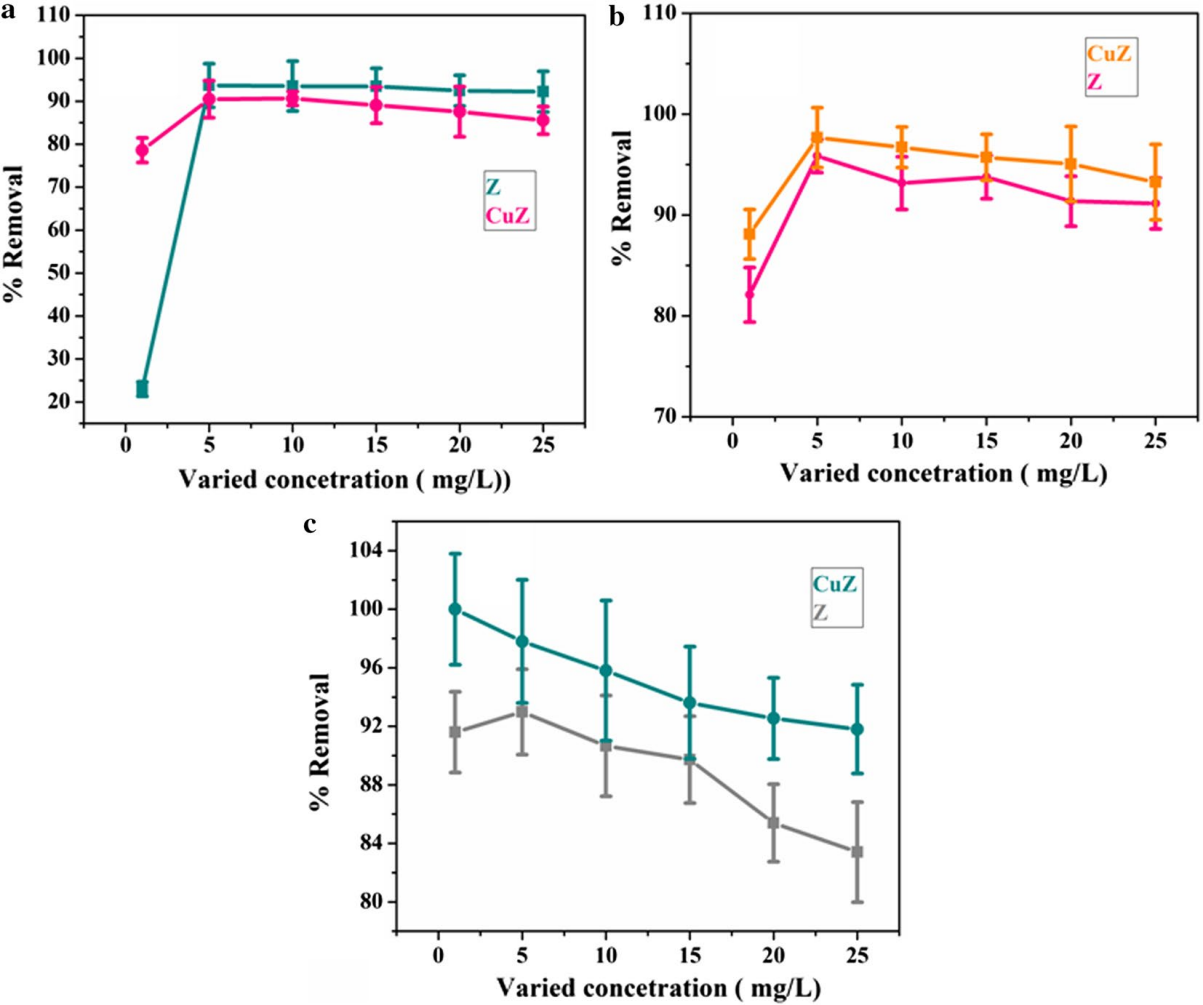
Effect of initial metal concentration on the % retention Pb (**a**), Cd (**b**) and Cr (**c**) by zeolite X and copper doped zeolite X

To investigate the effect of initial concentration on the removal efficiency of heavy metals, we also carried out intensive optimization experiments revealing the effect of initial metal concentration. To different 50 mL size Erlenmeyer’s flask, 25 mL sample of 1, 5, 10, 15, 20 and 25 mg/L concentrations of Pb, Cd and Cr were added. To each of the above metal concentration, optimum dose of zeolite (2 g, 1 g, 5 g) and copper doped zeolite (2 g, 1.5 g and 1 g) for Pb, Cd and Cr were added, and the mixture were mechanically stirred for their optimum time. After filtration the concentration of Pb, Cd and Cr were analyzed using atomic absorption spectroscopy. The effects of initial metal concentration on the % retention of Pb, Cd and Cr by zeolite X and copper doped zeolite X is shown in Fig. 5. As shown in Fig. 5, except for Cr, the percentage removal efficiency Pb and Cd increases with increasing metal concentration in the aqueous solutions, and reached the saturation point at higher initial concentrations. It is revealed that copper doped zeolite x exhibited efficient removal efficiency of Cd and Cr compared with undoped zeolite x while comparable response for Pb particularly at higher initial concentration. At the same initial concentration of each metal (i.e. 5 mg/L), the maximum retention of the zeolite X and copper doped zeolite X were found to 93.7, 95.9 and 93.0% and 90.7, 97.7 and 100% for Pb, Cd and Cr respectively.

The increase in adsorption or removal capabilities of the adsorbents with increasing in metal ions concentration could be explained by an acceleration of diffusion of metal ion into the adsorbents because of increase in concentration gradient with increase in initial metal ion concentration. As a result, the extent of each metal ion uptake by the adsorbents increases significantly with the increase of contact time. The slight decrease in the removal capacity or being remain constant after the saturation point of the adsorbents at higher initial metal ion concentration could be due to higher probability of collision between the adsorbent particle (zeolite X and copper doped zeolite X) and the metal ions; and decrease in the available active sites for adsorption due their saturation.

### Leaching test

We conducted leaching tests to further evaluate the stability of the adsorbent. The mass of copper dissolved in the leach liquor after adsorption experiment was determined by elemental analysis in comparison with the original one in the zeolite framework. After 1 h adsorption experiment a very negligible (0.78 ppb) amount of copper was detected in the leach liquor for the prepared copper doped zeolite powder (Table 4). A low amount of copper detection in the leach liquor after adsorption test confirms relatively a good adhesion of copper with the adsorbent material (i.e. zeolite).

**Table 4:** ICP-OES test

Sample	Concentration
Initial raw material (i.e. zeolite)	0.00 mg/L
Copper doped zeolite before adsorption experiment	80 mg/L
Copper doped zeolite leach liquor after adsorption experiment	0.78 µg/L

### Application of zeolite X and copper doped zeolite X into adsorption experiment (real water samples from Akaki river)

Lead, cadmium and chromium removal efficiency of the prepared adsorbents, zeolite X and copper doped zeolite X, were tested on real water samples under optimized conditions. As it is clearly seen from Table 5, the concentrations of Pb, Cd and Cr in the waste water samples before treatment were higher than the permissible level (0.696, 0.795 and 0.658 mg/L respectively for Pb, Cd and Cr). This concentration was reduced to BDL, 0.005 and 0.052 mg/L after the treatment with the adsorbent Zeolite X and BDL, 0.005 and BDL for samples treated with copper doped zeolite X, respectively. Zeolite X and zeolite X doped with optimum copper loading have similar removal efficiency for Pb and Cd. Whereas copper doped zeolite X has better removal efficiency of Cr than bare zeolite X.

**Table 5:** Pb, Cd and Cr removal from the real water samples (Akaki river) using zeolite X and copper doped zeolite X at optimized conditions

Metal	Concentration before treatment (mg/L)	After treatment with zeolite X		After treatment with copper doped zeolite X	
		Concentration (mg/L)	Removal efficiency	Concentration (mg/L)	Removal efficiency
Pb	0.696	BDL	100	BDL	100
Cd	0.795	0.005	99.37	0.005	99.37
Cr	0.658	0.052	92	BDL	100

### Microbial removal efficiency

**Fig. 6:**
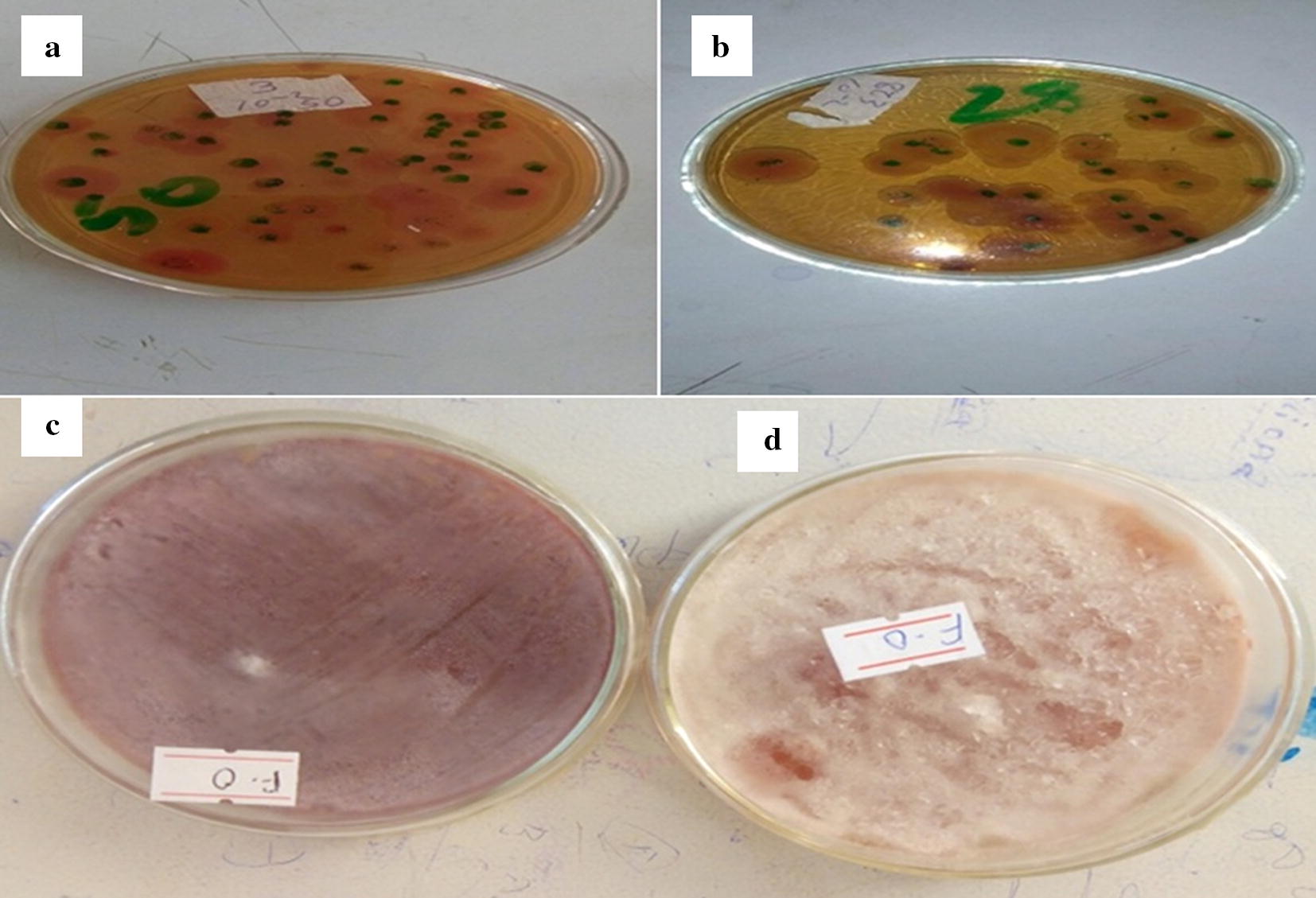
Total coliform counting after incubation of 2 days (48 h) of raw sample (**a**) and after treated with zeolite X (**c**); and before treated (**b**) and after treated with copper doped zeolite X (**d**)

As indicated in Table 6 and Fig. 6, the concentration of total coliform before treatment was 5.45 × 10^4^ CFU/100 mL and after treatment in the filtrates runs was zero (CFU) per 100 mL of sample water. This was however expressed in terms of percentage reduction of total coliform removal denoted the microbial removal efficiency of 100% both for zeolite X and copper doped zeolite X. This is most likely obtained due to high porosity of the adsorbent because of the high percentage of contact time. Zeolite X and copper-modified zeolite X enables completely eliminating microbes after 90 and 50 min contact time on average, demonstrating its effectiveness as a disinfectant respectively. Copper doped zeolite X with relatively high porosity and short contact time had good efficiency in removing microbial from bacterially contaminated water sources. When microorganism in contact with zeolite X and copper doped zeolite X, there might be strong suffocation on their path due to the tortuosity of the path, and at the same time they compete for feeding which reduce the number of microbials after treatment.

**Table 6:** Microbial removal efficiency of the adsorbents

Code	Sample	Total coliform (CUF)	% removal
A	Raw sample	5.45 × 10^4^ CFU/100 mL	–
B	Treated sample with zeolite X (at its optimum time)	0	100
C	Treated sample with copper doped zeolite X (at its optimum time)	0	100

### Properties of water samples before and after treatment

Table 7 shows the physico-chemical parameters data of the water sample before and after treatment. It can be concluded that most of the parameters were changed after treatment towards the safe limit set by WHO. The reduction of COD and BOD might be the organic compounds from the river’s wastewater trapped in the pores of zeolite and copper-doped zeolite adsorbents. Hence, the oxygen needed to oxidize the organic substances becomes minimal and subsequently the COD and BOD content decreased significantly.

**Table 7:** Properties of raw water before treatment and after treatment with adsorbents

Parameter	Raw sample (A)	Water sample treated with zeolite (B)	Water sample treated with copper doped zeolite (C)
Temperature (°C)	22.45	22.12	22.42
pH	7.301	7.5	7.5
Conductivity (μS/cm)	585	13.6	27.6
Turbidity (NTU)	78.6	4.7	4.2
DO (mg/L)			
Before incubation (average)	4.8	6.2	5.8
After incubation (average)	2.658	5.426	5.146
BOD (mg/L)	22.14	7.74	6.54
COD (mg/L)	219.2	6.8	7

## Conclusions

The use of copper-doped synthetic zeolite and bare synthetic zeolite were investigated for their combined heavy metal removal, antibacterial activity and reduction of physical parameters (BOD, COD, turbidity and electrical conductivity). The synthesized materials were characterized by X-ray diffraction (XRD), Fourier-transform infrared radiation (FTIR), autosorb IQ automated gas sorption analyzer and multiple-point Brunauer–Emmett–Teller (BET) method. The overall experimental results show that both bare zeolite X and the copper-doped synthetic zeolite X exhibited good antibacterial properties and heavy metal as well as physical parameters removal efficiency. However, the later material showed complete removal of the heavy metals under the same condition and with complete elimination of bacteria within 90 and 50 min of contact time respectively when bare zeolite X and the copper-doped synthetic zeolite X used. Hence, this adsorbent has very good potentials for simultaneous removal of microbes, organic maters and toxic metals removal from high-volume contaminated wastewaters. It also provides a substitute for the use of other expensive and less efficient materials as adsorbent.

## Abbreviations

FTIR: Fourier-transform infrared spectroscopy
DO: dissolved oxygen
BOD: biochemical oxygen demand
COD: chemical oxygen demand
XRD: X-ray diffraction
Z: zeolite
CUZ: copper doped zeolites
BET: Brunauer–Emmett–Teller
MDL: method detection limit
BDL: bellow detection limit
ICP-OES: inductively coupled plasma optical emission spectrometry
